# Comparison of Target Recognition by TRAF1 and TRAF2

**DOI:** 10.3390/ijms21082895

**Published:** 2020-04-21

**Authors:** Chang Min Kim, Hyun Ho Park

**Affiliations:** College of Pharmacy, Chung-Ang University, Dongjag-gu, Seoul 06974, Korea; 6427372@naver.com

**Keywords:** apoptosis, inflammation, TRAF, TRADD, protein interaction

## Abstract

Although TRAF1 and TRAF2 share common receptors and have extremely conserved amino acid residues, recent studies have shown that key differences in receptor binding preferences with different affinities exist, which might be important for their different functions in TRAF-mediated signal transduction. To better understand TRAF1 and TRAF2 signaling, we analyzed and compared their receptor binding-affinities. Our study revealed that TRADD, TANK, and caspase-2 bind to both TRAF1 and TRAF2 with different affinities in vitro. Sequence and structural analyses revealed that S454 on TRAF2 (corresponding to A369 of TRAF1) is critical for the binding of TRADD, and F347 on TRAF1 (corresponding to L432 of TRAF2) is a critical determinant for high affinity binding of TANK and caspase-2.

## 1. Introduction

Tumor necrosis factor receptor-associated factors (TRAFs) are important intracellular adaptor signaling molecules that mediate apoptosis and immune cell signaling [1,2,3]. TRAFs have a scaffolding activity, which can mediate extracellular and intracellular signaling. The TRAF domain, which is the protein interacting domain, interacts with various receptors as well as intracellular signaling molecules [4,5]. Although seven TRAF proteins have been identified in mammals, TRAF1-TRAF6 are considered true TRAF proteins, containing a TRAF domain, which is a feature of these molecules [3,5]. In addition to having scaffolding activity, TRAFs, with the exception of TRAF1, have an E3 ubiquitin ligase activity, containing an N-terminal RING finger domain, a common feature of E3 ubiquitin ligases [6,7,8]. Both the scaffolding and E3 ubiquitin ligase activities of TRAFs are critical for nuclear factor-κB (NF-κB), mitogen-activated protein kinases (MAPKs), and interferon-regulatory factors (IRFs) activation, playing pivotal roles in the regulation of inflammation and innate immunity, tissue homeostasis, stress responses, and apoptosis. As such, TRAFs are linked to many human diseases, including cancers and immune disorders [1,9,10,11,12].

Among the TRAFs, TRAF2 is the most intensively studied family member. TRAF2 is particularly important for TNF-α-mediated activation of MAPK/c-Jun N-terminal kinase (JNK) and NF-κB [1,13,14,15]. TRAF2 performs its function by interacting with several receptors including CD40, CD30, tumor necrosis factor receptor 2 (TNFR 2), Ox40, TRAF interacting protein (TRIP), and receptor activator of NF-κB (RANK) along with various other intracellular signaling molecules including TRAF-associated NF-kB activator (TANK) and caspase-2 [14,16,17,18]. The tumor necrosis factor receptor type 1-associated death domain protein (TRADD), a TNF-receptor associated apoptotic signal transducer, is also a TRAF2-binding death-domain containing adaptor molecule that mediates interactions between TNFR and TRAF, either for activation or inhibition of apoptosis [19,20,21].

TRAF1 is highly homologous to TRAF2, sharing ~70% sequence identity [22]. TRAF1, however, is considered unique in that it does not have a RING domain, indicating that TRAF1 is not an E3 ubiquitin ligase [23]. Even without E3 ubiquitin ligase activity, anti- and pro-apoptotic functions of TRAF1 have been discovered in immune and neuronal cells, respectively [24,25]. The role of TRAF1 as a positive regulator in insulin resistance and hepatic steatosis was also recently highlighted [26].

**Figure 1:**
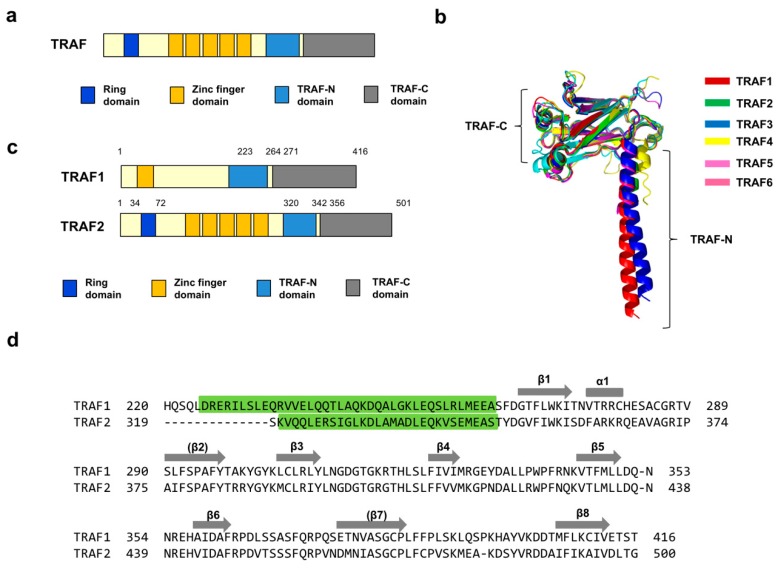
Domain organization and sequence comparison between TRAF1 and TRAF2. (**a**) General domains of the TRAF family. (**b**) Structural comparison of the TRAF family by superposition. All known structures of the TRAF domain from each TRAF member were superimposed using Pymol. (**c**) Domains of TRAF1 and TRAF2. (**d**) Sequence comparison of the TRAF domain between TRAF1 and TRAF2. Green highlighted and un-highlighted parts indicate TRAF-N and TRAF-C, respectively. Secondary structures are shown above the corresponding residues.

All TRAFs, except TRAF7, contain a protein-interacting domain, the TRAF domain, at their C-terminus, which is divided into two distinct regions, a TRAF-N coiled-coil and TRAF-C globular subdomain (Figure 1a). Structural studies of the TRAF domains of TRAF1 [27], TRAF2 [28], TRAF3 [29], TRAF4 [30,31], TRAF5 [29], and TRAF6 [2] have revealed that the globular subdomain is composed of seven to eight anti-parallel β-sheet folds (Figure 1b). The functional organization of the TRAF domains comprise mushroom-like trimeric structures [3]. Despite their sequence and structural similarities, each TRAF has its own binding-partner specificity. Although TRAF4 and TRAF6 recognize completely different sequences, it is well established that TRAF1-3 and 5 share almost the same binding motifs. Three motifs, Px(Q/E)E, Px(Q/E)xxD, and Px(Q/E)xT, are characterized as TRAF1-3 and 5 binding motifs. 

Despite sequence and structural similarities between TRAF1 and TRAF2, and although they share various receptors, their functional roles in the cell are different. Therefore, to better understand TRAF1- and TRAF2-mediated signaling, we characterized and compared the two TRAF-domain receptor (adaptor)-binding pockets of TRAF1 and TRAF2 (the co-receptor binding and TRADD adaptor binding pockets) by quantitative affinity analyses and structural comparisons. Our study revealed that TRADD, TANK, and caspase-2 bind to both TRAF1 and TRAF2, albeit with different affinities. TRADD interacts more strongly with TRAF2, while TANK and caspase-2 interact more strongly with TRAF1. Sequence and structural analyses revealed that S454 on TRAF2 (corresponding to A369 on TRAF1) is critical for binding to TRADD, and F347 on TRAF1 (corresponding to L432 on TRAF2) is the critical determinant for high-affinity interactions to signaling molecules, including TANK and caspase-2. 

## 2. Results

### 2.1. Both TRAF1 and TRAF2 Interact with TRADD via Their TRAF Domain In Vitro

TRAF1 and TRAF2 play distinct roles in the cell even though they share various binding partners. Unlike TRAF2 and other TRAF family members, TRAF1 does not contain an N-terminal RING domain (Figure 1c). To better understand TRAF1- and TRAF2-mediated signaling and their functional differences, we compared the two TRAF-domain receptor-binding pockets by sequence alignment. This analysis revealed that the amino acid sequences of the two well-known receptor-binding pockets of TRAF1 and TRAF2 are highly homologous, sharing ~70% sequence identity (Figure 1d).

**Figure 2:**
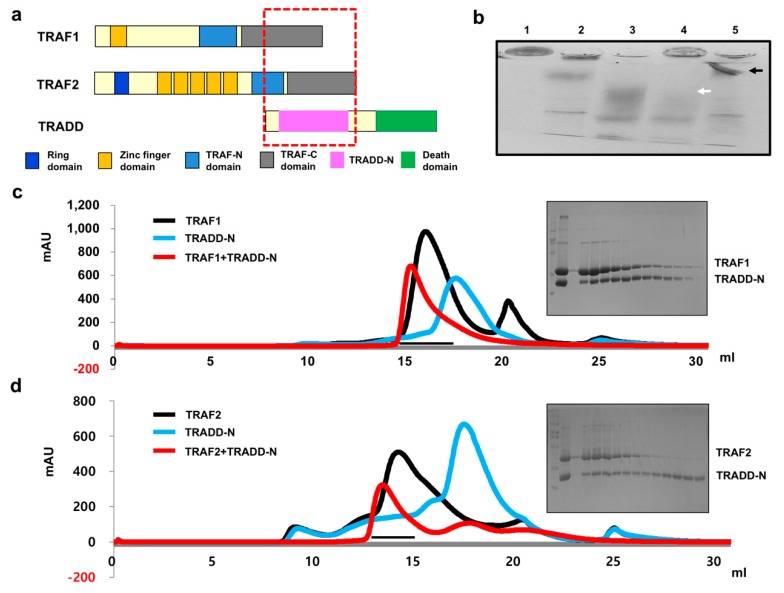
In vitro TRADD interactions between TRAF1 and TRAF2. (**a**) Schematic diagrams of the domain compositions of TRAF1 and TRAF2 with their tentative binding partner, TRADD. Binding region is indicated by red dotted box. (**b**) Native-PAGE. Lane1: TRAF1, Lane2: TRAF2, Lane3: TRADD-N, Lane4: TRAF1+TRADD-N, Lane5: TRAF2+TRADD-N. The white arrow indicates the position of the disappearance of TRADD-N due to complex formation. The black arrow indicates a newly produced band due to complex formation. (**c**,**d**) Size-exclusion chromatography profiles. Elution profiles of protein-complex peaks generated by mixing TRAF1 and TRADD-N (**c**) and TRAF2 and TRADD-N (**d**) and fractions loaded on SDS-PAGE (shown at the right part from the peaks). Loaded factions are indicated by black bar.

TRADD is a well-known regulator of the TRAF function. The death domain located at the C-terminus of TRADD interacts with death-domain containing receptors and adaptor proteins including TNFR, receptor interacting protein 1 (RIP1) and Fas-associated death domain protein (FADD), while the N-terminal domain of TRADD (TRADD-N) interacts with the TRAF domain of TRAF2 (Figure 2a) [21,32]. Although several indirect interactions between TRAF1 and TRADD have been described [20], a direct interaction and direct analyses between TRAF1 and TRADD have not been reported. To directly study their interactions and binding properties, which may provide important clues for understanding TRAF-mediated signaling by TRADD, TRAFs and TRADD-N were purified, and initial interaction testing was performed by Native-PAGE. Our experimental data showed that the TRADD-N band was completely absent, forming a new complex band when it was incubated with TRAF2, while only a small portion of the TRADD-N band disappeared when incubated with TRAF1, indicating that TRADD-N tightly interacts with TRAF2. Although TRAF1 did interact with TRADD-N, this interaction was not as tight as with TRAF2 (Figure 2b). To further confirm this association, the interaction between the TRAFs and TRADD-N was further tested by size-exclusion chromatography. Purified TRAF1 and TRAF2 were mixed with TRADD-N and incubated for 1 h before being subjected to size-exclusion chromatography. A shift in the eluted peak, approximately 16 mL in the TRAF1 and TRADD-N mixture, suggests that TRAF1 may form a complex with TRADD-N (Figure 2c). Interestingly, SDS-PAGE analysis of the eluted peak showed that TRADD-N did not perfectly co-migrate with TRAF1, indicating that TRADD-N did not bind all three subunits of trimeric TRAF1 in solution. This may be due to the low affinity of TRADD-N to TRAF1. In contrast, the mixture of TRAF2 and TRADD-N clearly caused a shift in the elution peak profile, which eluted around 14 mL (Figure 2d) and co-migrated well with TRADD-N at similar intensities by SDS-PAGE analysis (Figure 2d), indicating that the TRAF2 interaction with TRADD-N is tight and that the three subunits of TRAF2 may be occupied by TRADD-N.

### 2.2. TRADD Interacts with TRAF2 with Higher Affinity than TRAF1

**Figure 3:**
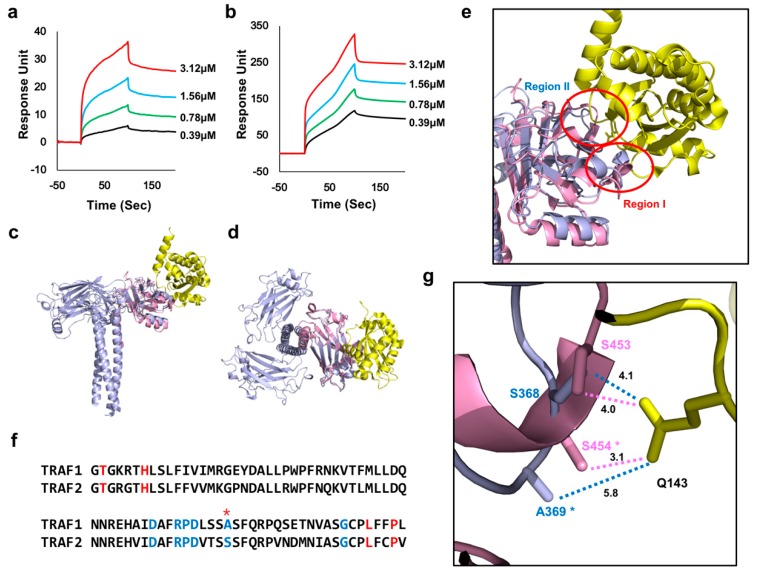
Quantitative affinity and structure analyses of the interaction between TRAFs and TRADD. (**a**,**b**) SPR characterization of the TRADD-N interaction with TRAF1 (**a**) and TRAF2 (**b**). TRADD-N was applied to TRAF1- and TRAF2-coupled sensor chips. (**c**,**d**) Structural models of TRAF1:TRADD-N complexes generated by the structural information of the previously solved TRAF2:TRADD-N complex. Light blue and pink colors indicate TRAF2 and TRAF1, respectively. Yellow color indicates TRADD-N that binds to TRAF2. Side view (**c**) and top view (**d**) are shown. (**e**) Two TRADD interacting regions on TRAF2. (**f**) Sequence alignment of the TRADD binding regions of TRAF1 and TRAF2. Red and blue colors indicate the TRADD interacting residues of TRAF1 and TRAF2 at Regions I and II, respectively. The red star indicates the amino acid residue that differs among the residues involved in the TRADD interaction. (**g**) Close-up view of the Region I interaction interface. Light blue and pink colors indicate TRAF2 and TRAF1, respectively. Yellow color indicates TRADD-N. The star marks the residues that differ among residues that are involved in the TRADD interaction. The interactions and distances are indicated by dashed line and number, respectively. The units of distance are in Å.

The binding affinities between TRADD-N and TRAF1 or TRAF2 were further quantitatively analyzed using surface plasmon resonance (SPR) experiments (Figure 3a,b). For the SPR experiment, each purified 6xHis-tagged TRAF1 and TRAF2 was coupled to Ni-NTA sensor chips using a His-tag affinity system. Different concentrations of TRADD-N protein, from 0.39 to 3.125 μM, were analyzed for binding to TRAF1 or TRAF2. Based on the clear concentration-dependent interaction SPR patterns, affinity parameters were calculated and the dissociation constant (Kd) values for TRADD-N interaction to TRAF1 and TRAF2 were 82 and 18 nM, respectively. The SPR values indicated that the affinity of TRAF2 for TRADD-N was four times higher than that of TRAF1. Given their interaction patterns and affinity differences in complex formation, we modeled the structure of the TRAF1:TRADD-N complex by docking TRADD-N to a previously solved TRAF1 structure based on the TRAF2:TRADD-N complex to better understand the affinity differences (Figure 3c,d). According to the previously determined structure of the TRAF2:TRADD-N complex, two distinct binding regions, Region I and II, were identified (Figure 3e) [21]. The specific residues from TRAF2 that contributed to the interaction in Region I were T401, H406, L471, and P474. The interaction-contributing residues on TRAF2 in Region II were D445, R448, P449, D450, S454 and G468. All residues in TRAF2 that participated in the interaction with TRADD-N were conserved in TRAF1, except for S454, which was replaced by alanine in TRAF1 (Figure 3f). The side chain of S454 in TRAF2 formed a stable hydrogen bond with the side chain of Q143 on TRADD-N and contributed to the stable interaction between these two proteins. The corresponding S454 residue, A369 on TRAF1, was located 5.8 Å away from Q143 on TRADD-N, making A369 unable to contribute to the interaction with TRADD-N (Figure 3g). To firmly demonstrate the role of S454 of TRAF2 in its more efficient TRADD recognition, we generated S454A mutant and analysis the interaction with TRADD via ITC. ITC analysis showed that the TRADD binding affinity to TRAF2 S454A mutant was dropped to Kd value of around 55 nM, which is similar Kd value with the TRADD binding affinity to TRAF1 (Supplementary Figure S1). 

Based on SPR and structure analyses, we concluded that TRADD-N interacts with TRAF2 more strongly than TRAF1, and that S454 on TRAF2 positively contributes to the interaction with TRADD, resulting in a higher binding affinity to TRADD-N.

### 2.3. TANK and Caspase-2 Interact more Tightly with TRAF1 than TRAF2

**Figure 4:**
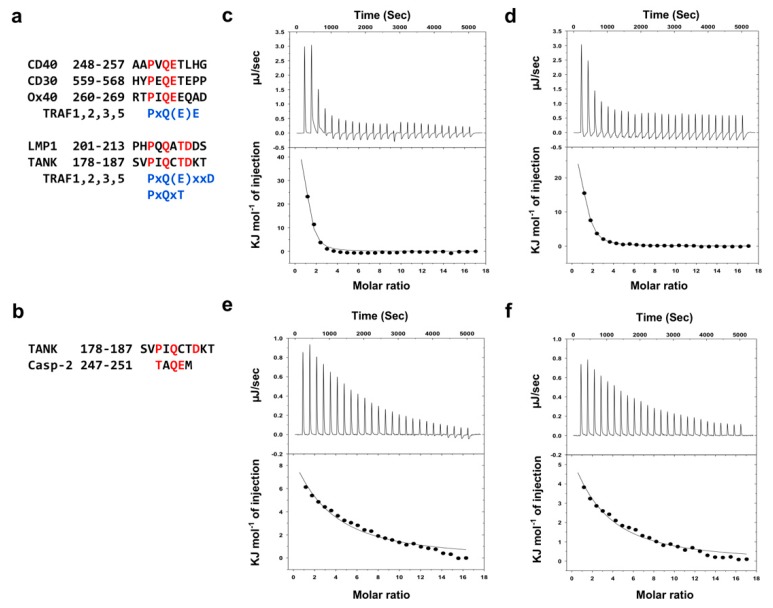
Quantitative interaction analyses of TANK and caspase-2 on TRAF1 and TRAF2. (**a**) TRAF1- and TRAF2-binding motif. Major motif Px(Q/E)E and minor motifs Px(Q/E)xxD or PxQxT are shown in the upper and lower panels, respectively. (**b**) Peptide sequences of TANK and caspase-2 that are used in the interaction study and contain TRAF1- and TRAF2-binding motifs, indicated as red color. (**c**–**f**) Isothermal titration calorimetry (ITC) used to assess peptide-protein interactions. The TANK peptide was titrated into a TRAF1 solution (**c**) and TRAF2 solution (**d**). Additionally, the caspase-2 peptide was titrated into a TRAF1 (**e**) and TRAF2 solution (**f**). Experimental fitting of the data to a single site interaction model is shown. Calorimetric titration and fitting data are shown in the upper and lower panels, respectively. The data presented are the average from two independent experiments.

In addition to TRADD, TANK and caspase-2 are known to be TRAF1- and TRAF2-binidng proteins [14,17,33]. As the sequences of the binding hot spots are conserved in TRAF1 and TRAF2, they also share the same binding consensus motifs, namely, a major motif, Px(Q/E)E, and two minor motifs, Px(Q/E)xxD and PxQxT (Figure 4a) [3]. Although possessing nearly identical receptor-binding sites, a previous deep mutational scanning study revealed key differences in the binding preferences and affinities between TRAF1 and TRAF2 [34]. In this regard, we wondered how TRAF1 and TRAF2 bind differently even though they share almost identical receptor-binding motifs. To better understand this, we analyzed the interactions between TRAF1 or TRAF2 and two known receptor peptides, TANK (SVPIQCTDKT) and caspase-2 (TAQEM). Quantitative TANK peptide interactions to TRAFs have been previously examined in a previous study [33]. TRAF interactions with the caspase-2 peptide, however, have not been quantitatively analyzed, although a recent study indicated that caspase-2, especially the region of TAQEM, directly interacts with TRAF2 [17]. Based on these preliminary binding studies, we thoroughly analyzed the interactions of the TANK and caspase-2 peptides with TRAF1 and TRAF2 using isothermal titration calorimetry (ITC). Both peptides (1 mM) were titrated with 20 μM of TRAF1 or TRAF2 with 25 injections for incremental ITC experiments (Figure 4c,d). In both cases, the reactions were endothermic, and the observed heat profiles were in agreement with ideal interaction values, indicating the existence of a single binding site without distinct cooperativity in the interaction. The Kd values for TANK were 2.5 μM for TRAF1 (Figure 4c) and 11.7 μM for TRAF2 (Figure 4d), which indicates that the TANK peptide interacts more tightly with TRAF1 and with approximately five times higher affinity. The Kd values of the caspase-2 peptide were 86.9 μM for TRAF1 (Figure 4e) and 116.7 μM for TRAF2 (Figure 4f), indicating that caspase-2 also interacts more tightly with TRAF1 than with TRAF2, although the binding affinity is much lower compared to that of the TANK peptide.

### 2.4. F347 on TRAF1 Positively Effects the Affinity of TANK and Caspase-2

**Figure 5:**
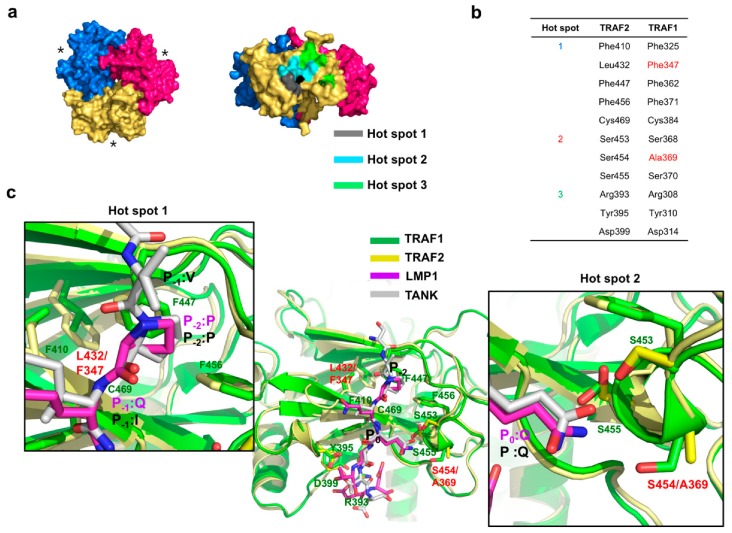
Structure analyses of the interaction between TRAFs and receptors, including TANK and caspase-2. (**a**) Prototype trimeric structure of the TRAF-C domain of TRAFs. Three domains are shown with different colors. Receptor-interaction regions are marked with black stars. Receptor binding hot spots are indicated. Top and side views are shown in the left and right panels, respectively. (**b**) Receptor-binding hot spots and conserved amino acid residues in TRAF1 and TRAF2 which are involved in the interaction with various receptors. The amino acid residues that are not conserved are colored in red. (**c**) Comparison of the receptor-interacting region between TRAF1 and TRAF2. The structure of TRAF1 (yellow):TANK (gray peptide) complex was compared with the structure of TRAF2 (green):LMP1 (magenta peptide) complex by structural superposition. All the amino acid residues in TRAF2 involved in the interaction with the LMP1 peptide are labelled in green. Non-conserved amino acid residues are labelled in red. Amino acid positions of the TRAF-binding motif in the receptor peptides are labeled as P-2 and P0. Close-up views of binding hot spot1 and hot spot2 are shown in the left and right panels, respectively.

To understand how TRAF1 binds with higher affinity to TANK and caspase-2, we compared the amino acid sequence of the receptor-binding hot spots on TRAF1 and TRAF2. There are three well-known hot spots in TRAFs (Figure 5a). Based on the sequence comparison, two amino acid residues in TRAF1, F347 of hot spot1 and A369 of hot spot2, were not conserved in TRAF2 (Figure 5b). As the two non-conserved amino acid residues of TRAF1 may generate different conditions for receptor-binding regions and affect its affinity for various receptors, we compared the receptor-binding regions between TRAF1 and TRAF2 by comparing the TRAF1:TANK structure with the TRAF2:latent membrane protein 1 (LMP1) structure (Figure 5c). This structural comparison showed that F347 on TRAF1 (corresponding to L432 on TRAF2) is critical for the formation of a hydrophobic pocket (hot spot1) to accommodate the P-2 position of receptor peptides. F347 might form a more stable hydrophobic pocket than leucine by being closer to other hydrophobic residues that are involved in the formation of hot spot1. A369 on TRAF1 (corresponding to S454 on TRAF2) did not contribute to the formation of a hydrogen-bond (H-bond) cluster with Q at the P0 position of receptor peptides. S454 of TRAF2 was not critical for the formation of an H-bond cluster, unlike residues S453 and S455, which were closely located to the Q at the P0 position of the receptor peptide. These structural analyses indicated that the sequence differences of S454 on TRAF2 and A369 on TRAF1 did not affect receptor preference and binding affinity. However, F347 on TRAF1 (corresponding to L432 on TRAF2) was critical for determining receptor preference and binding affinity by contributing to the formation of a stable hydrophobic pocket in the hot spot1 motif. To firmly demonstrate the role of F347 of TRAF1 in its more efficient receptors recognition, we generated TRAF1 F347L mutant and analysis the interaction with TANK and caspase-2 via ITC. ITC analysis showed that the TANK and casaspe-2 binding affinity to TRAF1 F347L mutant was dropped to Kd value of around 15 uM for TANK and 265 uM for caspase2, which are similar Kd value with the TANK and caspase-2 binding affinity to TRAF2 (Supplementary Figure S2a,b), indicating that F347 on TRAF1 positively contributes to the both TANK and caspase-2 interactions.

## 3. Discussion

Seven TRAF family members have been identified in mammals and each member performs distinct functions, especially in immune cell signaling. Although containing nearly identical receptor-binding environments composed of extremely conserved amino acid residues, especially TRAF1, TRAF2, TRAF3, and TRAF5, a recent study showed that key differences in binding preferences with different affinities exist, which might be important for TRAF-mediated signal transduction [34]. To understand how TRAF1 and TRAF2 have adaptor and receptor preferences with different affinities—even though they share almost identical binding motifs—we analyzed and compared adaptor (TRADD) and receptor (TANK and caspase-2) affinities for TRAF1 and TRAF2. We describe key differences that might be critical for determining adaptor and receptor-binding affinities and preferences by structural analysis. 

TRADD-N was used for binding-affinity comparisons on TRAF1 and TRAF2. Results of SPR analyses showed that TRADD-N interacted more strongly to TRAF2 that TRAF1. To better understand this difference, we performed structural and sequence analyses and found that S454 on TRAF2 (corresponding to A369 on TRAF1) contributed positively to the interaction with TRADD, increasing the binding affinity of TRADD with TRAF2.

Initial structural studies of the TRAF family in complex with various receptor peptides unveiled one major, Px(Q/E)E motif, and two minor TRAF-binding motifs, Px(Q/E)xxD and PxQxT [3]. Because the most conserved amino acids in the TRAF-binding motif are near the zero position of the TRAF-binding motif (P0), the residues of the Px(Q/E)E motif were named as P (P-2), x (P-1), Q/E (P0), and E (P1). Three receptor-binding hot spots on TRAFs were identified from a previous structural study [3]. To recognize the Px(Q/E)E motif on the receptor, amino acid residues on hot spot1 (F410, L432, F447, F456, and C469) in TRAF2 make extensive van der Waal contacts with P at the P-2 site. To accommodate Q at the P0 position, hot spot2 is composed of a serine triad, S453, S454, and S455 in TRAF2, involved in the formation of hydrogen bonds with Q at position P0. 

TANK and caspase-2, which are known to bind to the receptor-binding pocket of TRAFs, were used for this study. According to ITC, both TANK and caspase-2 peptides bind more strongly to TRAF1 than TRAF2. To understand this affinity difference, we compared the amino acid sequence between TRAF1 and TRAF2. Although most of the amino acid residues in TRAF2, which are critical for the interaction with the Px(Q/E)E motif, are conserved in TRAF1, two amino residues, L432 and S454, are not conserved. L432 of TRAF2 in hot spot1 is replaced by F347 in TRAF1. In addition, S454 in the serine triad of TRAF2 is replaced by A369 in TRAF1. According to our structure analyses, among the serine triad residues, S454 of TRAF2 (A369 in TRAF1) has the least effect on Q interaction at the P0 position, while the formation of a stable hydrophobic pocket by F347 in TRAF1 (L432 of TRAF2) was critical for receptor interaction, affecting receptor affinity and preference.

## 4. Materials and Methods

### 4.1. Peptide Preparation

For preparing the TRAF1 and TRAF2 interacting minimal consensus motifs, two peptides, TANK (SVPIQCTDKT) and caspase-2 (TAQEM) were synthesized and purified by Peptron (Dae-jeon, South Korea).

### 4.2. Visualization of Structure Models

The structure alignments and protein figures in this study were prepared using Pymol. 

### 4.3. Sequence Alignment

The amino acid sequences of the TRAF domains of TRAF1 and TRAF2 were analyzed using Clustal Omega (http://www.ebi.ac.uk/Tools/msa/clustalo/).

### 4.4. Protein Expression and Purification 

The expression and purification of the TRAF domains have been previously described [27,33]. In brief, the TRAF1 TRAF domain (amino acid residues 220-416) and TRAF2 TRAF domain (amino acid residues 305-501) were cloned into the pET24a expression vector containing a C-terminal 6x-histidine tag. The expression plasmid constructs were transformed into BL21 (DE3) E. coli and grown in Luria-Bertani (LB) media at 37 °C. Target protein expression was induced by the addition of 0.25 mM isopropyl β-D-thiogalactopyranoside (IPTG) for 20 h at 20 °C. The bacteria were pelleted, resuspended, and lysed by sonication. After clarifying the lysates by centrifugation, the expressed target proteins were purified by affinity chromatography using a gravity-flow column (Bio-Rad, Hercules, CA, USA) packed with 2 mL of Ni-NTA affinity resin (Qiagen, Hilden, Germany), and 100 mL of washing buffer (20 mM Tris-HCl pH 7.9, 500 mM NaCl, 60 mM imidazole) was used to remove unbound proteins. Each target protein was eluted with an imidazole-containing elution buffer (20 mM Tris-HCl pH 7.9, 500 mM NaCl, 250 mM imidazole). The purity was further improved by size-exclusion chromatography (SEC) using a Superdex 200 size-exclusion column 10/30 (GE healthcare) pre-equilibrated with a solution of 20 mM sodium citrate pH 5.0 and 1 M NaCl. 

For preparation of the N-terminal domain of TRADD (TRADD-N, amino acid residues 1-163), an expression construct was generated by cloning the fragment into a pET24a vector using NdeI and XhoI restriction sites. The purification of TRADD-N is almost identical to the method used for purifying TRAF1 and TRAF2, except for the SEC buffer in the final step (20 mM Tris-HCl pH 8.0 and 150 mM NaCl).

### 4.5. Native-PAGE

The formation of complexes was assayed by native (non-denaturing) PAGE conducted on a PhastSystem (GE Healthcare) with pre-cast 8% to 25% acrylamide gradient gels (GE Healthcare). Coomassie brilliant blue was used for the staining and detection of bands. TRADD-N was mixed with either TRAF1 or TRAF2 and incubated for 1 h at 4 °C, after which the mixture was subjected to electrophoresis. Complex formation was evaluated based on the appearance of newly formed bands or the disappearance of bands that were detected in single control protein bands. 

### 4.6. Complex Formation Assay by Gel-filtration Chromatography

Purified TRAF domains and TRADD-N proteins were mixed, incubated at 4 °C for 1 h, and concentrated to 4 to 5 mg/mL using a protein concentration kit (Millipore). The concentrated protein solutions were then applied to a Superdex 200 gel-filtration column 10/30 (GE healthcare) that had been pre-equilibrated with a solution of 20 mM Tris buffer at pH 8.0 and 150 mM NaCl. Complex assembly was evaluated based on the positions of eluted protein peaks monitored at 280 nm followed by SDS-PAGE.

### 4.7. Surface Plasmon Resonance (SPR)

For SPR experiments, a Biacore T200 (GE Healthcare) and NTA sensor chips were used. The sensor chip surface was coated by injecting with a 100 mM nickel solution. Then, each TRAF domain of TRAF1 or TRAF2 was diluted in PBS to a concentration of 100 μg/mL and injected at a rate of 10 μL/min for 1 min for tandem immobilization on the NTA chip surface. Concentrations ranging from 0.39 to 3.125 μM of TRADD-N were prepared by dilution in PBS and injected in the TRAF-domain-coated flow channel at a flowrate of 30 μL/min for 1 min, followed by a dissociation time of 300 s and regeneration with a mixture of EDTA and guanidine HCl. Raw sensorgrams were double blanked by subtracting responses from the reference flow channel and blank injection.

### 4.8. Isothermal Titration Calorimetry (ITC)

A NanoITC (TA Instruments) was used for isothermal titration calorimetry experiments. The TRAF domains of TRAF1 and TRAF2 were dialyzed extensively against PBS buffer, and the two peptides, TANK (SVPIQCTDKT) and caspase-2 (TAQEM), were dissolved in the same buffer to minimize heats of dilution. Prior to titration, all experimental samples were centrifuged at 13,000 rpm at 4 °C for 5 min to remove any debris. For each titration, a concentrated peptide solution (1 mM) was injected into a cell containing the TRAF1 or TRAF2 TRAF domain at a concentration of ~20 uM. All titrations were carried out at 15 °C with 25 injections at 160 s intervals. Binding isotherms were analyzed by using the software provided by TA Instruments. Baseline controls were acquired with buffer and pure peptide solutions.

## Supplementary Materials

Supplementary materials can be found at https://www.mdpi.com/1422-0067/21/8/2895/s1.
