# Supplementary material for: Comparison of Target Recognition by TRAF1 and TRAF2
Source: Int J Mol Sci. 2020 Apr 21;21(8):2895. doi: 10.3390/ijms21082895 (PMC7215387; doi:10.3390/ijms21082895)
Supplement: Supplementary file 1 [file ijms-21-02895-s001.pdf]

Supplementary Material

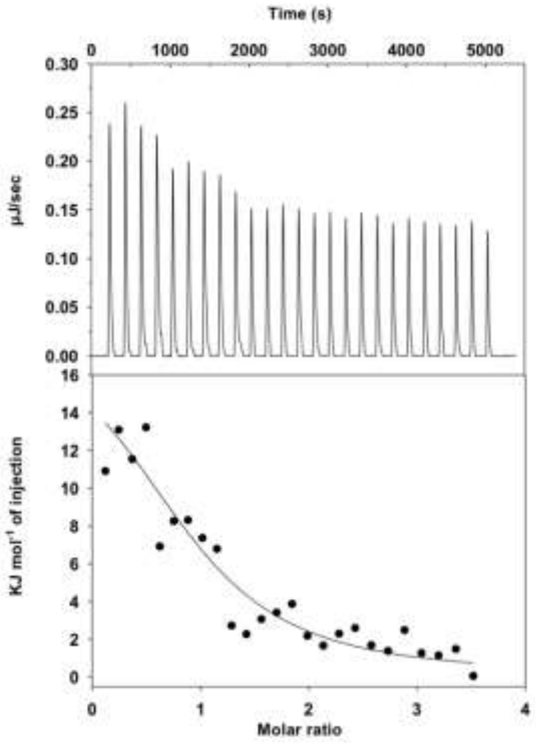

Supplementary Figure 1. Quantitative interaction analyses of TRADD on TRAF2 S454A mutant.

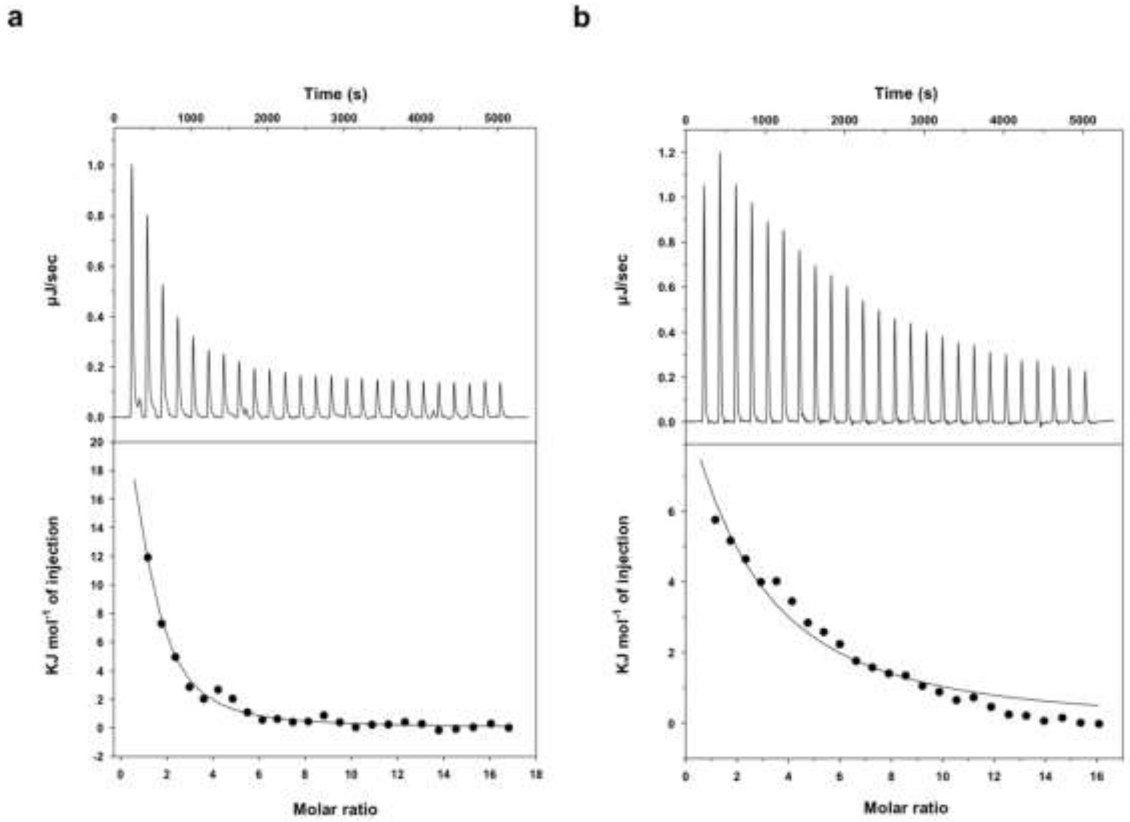

Supplementary Figure 2. Quantitative interaction analyses of TANK and caspase-2 on TRAF1 F357L mutant.
